# All-Cause and Cause-Specific Mortality among Users of Basal Insulins NPH, Detemir, and Glargine

**DOI:** 10.1371/journal.pone.0151910

**Published:** 2016-03-31

**Authors:** Arto Y. Strandberg, Fabian J. Hoti, Timo E. Strandberg, Solomon Christopher, Jari Haukka, Pasi Korhonen

**Affiliations:** 1 University of Helsinki, Clinicum, Helsinki, Finland; 2 Aava Medical Centre, Kerava, Finland; 3 EPID Research, Espoo, Finland; 4 Helsinki University Central Hospital, Helsinki, Finland; 5 University of Oulu, Center for Life Course Health Research, Oulu, Finland; University of Catanzaro Magna Graecia, ITALY

## Abstract

**Background:**

Insulin therapy in type 2 diabetes may increase mortality and cancer incidence, but the impact of different types of basal insulins on these endpoints is unclear. Compared to the traditional NPH insulin, the newer, longer-acting insulin analogues detemir and glargine have shown benefits in randomized controlled trials. Whether these advantages translate into lower mortality among users in real life is unknown.

**Objective:**

To estimate the differences in all-cause and cause-specific mortality rates between new users of basal insulins in a population-based study in Finland.

**Methods:**

23 751 individuals aged ≥40 with type 2 diabetes, who initiated basal insulin therapy in 2006–2009 were identified from national registers, with comprehensive data for mortality, causes of death, and background variables. Propensity score matching was performed on characteristics. Follow-up time was up to 4 years (median 1.7 years).

**Results:**

2078 deaths incurred. With NPH as reference, the adjusted HRs for all-cause mortality were 0.39 (95% CI, 0.30–0.50) for detemir, and 0.55 (95% CI, 0.44–0.69) for glargine. As compared to glargine, the HR was 0.71 (95% CI, 0.54–0.93) among detemir users. Compared to NPH, the mortality risk for both cardiovascular causes as well as cancer were also significantly lower for glargine, and especially for detemir in adjusted analysis. Furthermore, the results were robust in various sensitivity analyses.

**Conclusion:**

In real clinical practice, mortality was substantially higher among users of NPH insulin as compared to insulins detemir or glargine. Considering the large number of patients who require insulin therapy, this difference in risk may have major clinical and public health implications. Due to limitations of the observational study design, further investigation using an interventional study design is warranted.

## Introduction

The goal of insulin use in type 2 diabetes is to prevent microvascular and macrovascular complications associated with uncontrolled hyperglycemia. However, insulin therapy itself may increase the risk of cardiovascular events, cancer, and mortality [1–2], but the impact of different types of basal insulins on these endpoints is unclear.

In patients with type 2 diabetes basal insulin therapy is usually initiated with the conventional NPH (neutral protamine Hagedorn) insulin, or a newer, longer-acting basal insulin analogue detemir or glargine. NPH has been the predominant basal insulin in clinical use for several decades, but the use of glargine and detemir has gradually risen subsiding the use of NPH insulin. [3] However, the substantially higher cost of analogues (covered by patents) creates a financial burden on patients and insurers, and since in addition the superiority of newer analogues has not been proven [4], NPH insulin is recommended as the initial choice for insulin treatment for patients with type 2 diabetes in many countries.[5]

The three insulins differ in their molecular structure and pharmacokinetic action. Notably, compared to the peak absorption of 4–5 hours after injection of NPH [6], detemir and glargine have a relatively longer and peakless action [7–8]. Detemir and glargine have shown advantages, such as a lower risk of hypoglycaemia and weight gain, over NPH in randomized clinical trials (RCTs) [9–10], as well as in short observational studies [11]. However, it is not known whether these potential advantages translate into additional benefits on morbidity and mortality in the long term. RCTs are often of short duration, whereby hard endpoints such as cardiovascular and cancer mortality cannot be recognized. Moreover, the patients randomized into clinical trials are not necessarily representative of the general population, because patients with advanced age, co-morbidities, or a history of hypoglycaemias may have been excluded. On the other hand, in clinical practice the choice of insulin may be selective according to patient profile, and the type of insulin may be switched over time, blurring possible associations in retrospective studies. Overall, the mortality risk associated with insulin use has not been extensively examined in real-life, and well-designed studies for comparative safety between modern insulin types are needed.

The aim of this nationwide, register-based longitudinal study was to investigate the differences in the safety of insulins NPH, detemir, and glargine in terms of all-cause and cause-specific mortality among real-life patients with type 2 diabetes.

## Methods

### Ethics statement

This is a register-based study with anonymous data and no patient contact. The study protocol was approved by the Ethical Review Board of the Hjelt Institute, University of Helsinki Medical Faculty. The research permission numbers to use the data were obtained from the Social Insurance Institute (Kela 14/522/2011), the National Institute for Health and Welfare (Dnro THL/408/5.05.00/2011), and the Statistics Finland (TK-53-367-11).

### Study population

**Fig 1:**
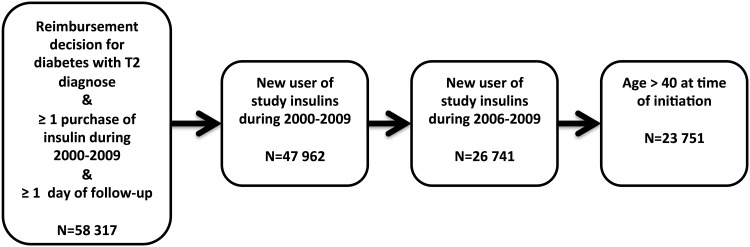
Study population flow chart.

The study population comprised 23 751 Finnish patients with type 2 diabetes, who at age 40 years and older had initiated therapy with basal insulin (NPH, detemir, or glargine) between January 1, 2006, and December 31, 2009 (Fig 1). The participants were insulin-naïve, i.e. they had no earlier prescriptions for basal insulins. During the study period, all three basal insulins were available, and they were 100% reimbursed for type 2 diabetes patients in Finland. Furthermore, these were the only basal insulins on the market and there were no recommendations regarding insulin preference for specific patient groups at that time.

### Sources of data

The patients were linked to nation-wide administrative registers through the unique personal identity code assigned to each citizen. In Finland, all individuals who have a prescription for drug treatment of diabetes are entitled to full reimbursement of medical expenses. This requires a detailed certificate from the treating physician. Expert physicians of the Social Insurance Institution of Finland (KELA) validate the certificates for diagnosis, and KELA maintains a register of the patients. All purchased and reimbursed medications are recorded in the Finnish Prescription Register (FPR) and Finnish Registry for Reimbursed Medication (FRM), with generic name, prescribed amount in defined daily doses (DDD) as defined by WHO, and date of purchase. The Finnish Hospital Care Register (FHCR), administered by the National Institute for Health and Welfare (THL), contains information on hospitalization events such as diagnosis (ICD codes), and dates of admission and discharge. Mortality data was retrieved from Statistics Finland (SF), where the vital status is collected for all Finnish citizens into the Finnish Causes of Death Register (FCDR)—irrespective whether they die in Finland or abroad. [12] Consequently, the mortality coverage of the follow-up was virtually complete. [13] These same registers have been utilized in several recent diabetes, [14–15] and mortality related studies [16].

Using these registers we were able to identify practically every patient with type 2 diabetes, who during the study period was entitled to reimbursement of insulin (Category 103 with ICD-10 International Classification of Diseases—the 10th revision [ICD-10] diagnosis code E11) in Finland. The three basal insulins were compared for all-cause and cause-specific mortality. FHCR was used to gather data on hospitalizations due to severe hypoglycaemia (ICD-10 diagnosis code E10.00 and E11.00). Prior and current use of insulin and sulfonylurea was obtained from the FPR, based on purchase information since 1995. Information on hospitalizations due to other reasons, or information on the use of other medication were not obtained.

### Statistical methods

The index date was defined as the date of the first purchase of a prescribed basal insulin. Drug exposure periods started at the date of purchase, and the length of the exposure period was estimated according to the number of DDD’s and the average daily dosage, as contained in the purchases over the entire follow-up. To avoid introducing gaps between consecutive drug exposure periods, a 15% grace period was added to the length of the underlying purchase. The time dependent exposure variable, “current use of NPH, detemir, or glargine”, was based on these exposure periods. In case of a switch between the basal insulins we assumed that the patient had the most recent insulin in use.

Any follow-up time that was not covered by the exposure periods of the three insulins was assigned to a category”Unknown”. The deaths which occurred during this time were not accounted for in any basal insulin group but were analysed separately as a group of “Unknown deaths”.

### Propensity score matching

To reduce potential selection bias, and to balance the three insulin groups, PS matching technique [17] was used to form a subcohort (N = 9 363) with 3 121 patients in each basal insulin group. Propensity scores [18]—the probabilities to initiate insulin NPH, detemir and glargine—were calculated for each participant conditional on the following covariates: Age at baseline (10-year age groups), gender, prior use of non-basal insulin (yes/no), prior use of sulpholnylureas (yes/no), prior hospitalization due to severe hypoglycaemia (yes/no), and years from diagnosis of diabetes at the index date. These variables could potentially influence the selection of insulin type by the attending physician. For each patient, PS were calculated separately within each calendar year of cohort entry (2006–2009). The treatment groups were then balanced across these covariates in a 1:1:1 ratio by selecting the group with the smallest number of initiators, and then matching persons (according to the closest PS) were picked from the two comparator groups. We used Euclidean distance in the 3-dimensional space spanned by the three PS to identify the closest matches.

Kaplan-Meier curves for survival were estimated for the cohorts prior to and after matching, and Cox's proportional hazards (PH) models were used to calculate adjusted hazard ratios (HRs) with 95% confidence intervals [CI]) for mortality associated with insulin use. For all-cause mortality analysis P values of less than 0.05 were considered statistically significant. For cause-specific analyses Bonferroni correction was applied. As matching only assures balance in PS variables between treatment groups at baseline, the Cox’s PH model was adjusted for all PS-variables as well as for the time dependent variables “current use of non-basal insulin”, “current use of sulfonylurea”, and “switch of basal insulin”. The PH assumption for basal insulin, age and gender was examined by plotting the stratified Kaplan-Meier curves. R software was used for the analyses.

### Sensitivity analyses

To address potential bias due to unknown exposure to basal insulin we performed two sensitivity analyses. In the first, follow-up periods of unknown exposure were censored, and in the second, we assumed that patients continued the use of the most recent basal insulin during the periods of unknown exposure. In the main sensitivity analysis we did not exclude persons who had previous exposure to non-basal insulin, because these consisted mostly of brief use of a short acting insulin during periods of ill health or pregnancy. To address this potential bias we performed a sensitivity analysis where patients with previous use of non-basal insulin were excluded. In the outcome analysis, switching between basal insulins was adjusted for by including a time dependent indicator variable of the first switch in insulin regimen. Furthermore sensitivity analyses where follow-up was censored at the time of the first switch was performed, and also where follow-up time periods of overlapping use of basal insulin were censored. (See S1 Table)

## Results

### Study patients

Between January 1, 2006 and December 31, 2009, there were 23 751 patients with type 2 diabetes, who initiated use of NPH (N = 8535, 35.9%), detemir (N = 4749, 20.0%), or glargine (N = 10 467, 44.1%), at age 40 or over (Fig 1). Mean age in the total cohort was 65.5 years. 58% were male. Prior to matching, the baseline distributions of gender and age were similar between insulin groups, although there were slightly less initiators of detemir in the older age groups (70–79 and >79 years) (Table 1).

**Table 1:** Baseline characteristics for the three basal insulin initializers prior to matching and for the propensity score matched cohort.

Baseline* variable	Unmatched cohort, N = 23751							Propensity score matched cohort, N = 9363						
	Insulin NPH, N = 8535		Detemir, N = 4749		Glargine, N = 10467			Insulin NPH, N = 3121		Detemir, N = 3121		Glargine, N = 3121		
	no.	%	no.	%	no.	%	P-value^1^	no.	%	no.	%	no.	%	P-value^1^
Gender														
Male	4977	58.3	2773	58.4	6158	58.8	0.8	1808	57.9	1823	58.4	1823	58.4	0.9
Female	3558	41.7	1976	41.6	4309	41.2		1313	42.1	1298	41.6	1298	41.6	
Age, years														
40–49	771	9.0	506	10.7	975	9.3	<0.001	299	9.6	288	9.2	287	9.2	1.0
50–59	1965	23.0	1231	25.9	2448	23.4		703	22.5	735	23.6	722	23.1	
60–69	2423	28.4	1479	31.1	2918	27.9		903	28.9	890	28.5	899	28.8	
70–79	2077	24.3	1000	21.1	2474	23.6		716	22.9	727	23.3	724	23.2	
80+	1299	15.2	533	11.2	1652	15.8		500	16.0	481	15.4	489	15.7	
Prior use of sulpholnylurea														
No	2919	34.2	1653	34.8	3290	31.4	<0.001	1190	38.1	1196	38.3	1195	38.3	1.0
Yes	5616	65.8	3096	65.2	7177	68.6		1931	61.9	1925	61.7	1926	61.7	
Prior use of non- basal insulin														
No	8154	95.5	4396	92.6	9898	94.6	<0.001	2941	94.2	2955	94.7	2950	94.5	0.7
Yes	381	4.5	353	7.4	569	5.4		180	5.8	166	5.3	171	5.5	
Prior hospitalizations due to severe hypoglycaemia														
No	7808	91.5	4428	93.2	9587	91.6	<0.001	2882	92.3	2908	93.2	2912	93.3	0.3
Yes	727	8.5	321	6.8	880	8.4		239	7.7	213	6.8	209	6.7	
Time since type 2 diabetes diagnosis														
≤1	2376	27.8	1077	22.7	2394	22.9	<0.001	924	29.6	933	29.9	925	29.6	0.8
>1-≤2	506	5.9	324	6.8	630	6.0		190	6.1	175	5.61	170	5.45	
>2-≤5	1917	22.5	1069	22.5	2239	21.4		642	20.6	659	21.12	686	21.98	
>5-≤10	2745	32.2	1671	35.2	3645	34.8		966	31.0	930	29.8	936	29.99	
>10-	991	11.6	608	12.8	1559	14.9		399	12.8	424	13.59	404	12.94	
Calendar year at start of follow-up														
2006	3657	42.9	55	1.2	451	4.3	<0.001	55	1.8	55	1.8	55	1.8	1.0
2007	2544	29.8	732	15.4	2595	24.8		732	23.5	732	23.5	732	23.5	
2008	1433	16.8	1684	35.5	3718	35.5		1433	45.9	1433	45.9	1433	45.9	
2009	901	10.6	2278	48.0	3703	35.4		901	28.9	901	28.9	901	28.9	

* Baseline is the date of purchase of first basal insulin.

^1^P-value based on Pearson’s Chi-Square test

For other variables there were no major differences except for the calendar year of start of the first insulin: The number of prescriptions for NPH declined during the follow up, whereas there was a rising trend for prescriptions for detemir and glargine with time. To reduce the potential bias, these covariates have been taken into account in the propensity score model. After PS matching, these and also other differences between the insulin groups were evened out (Table 1).

### All-cause mortality

**Fig 2:**
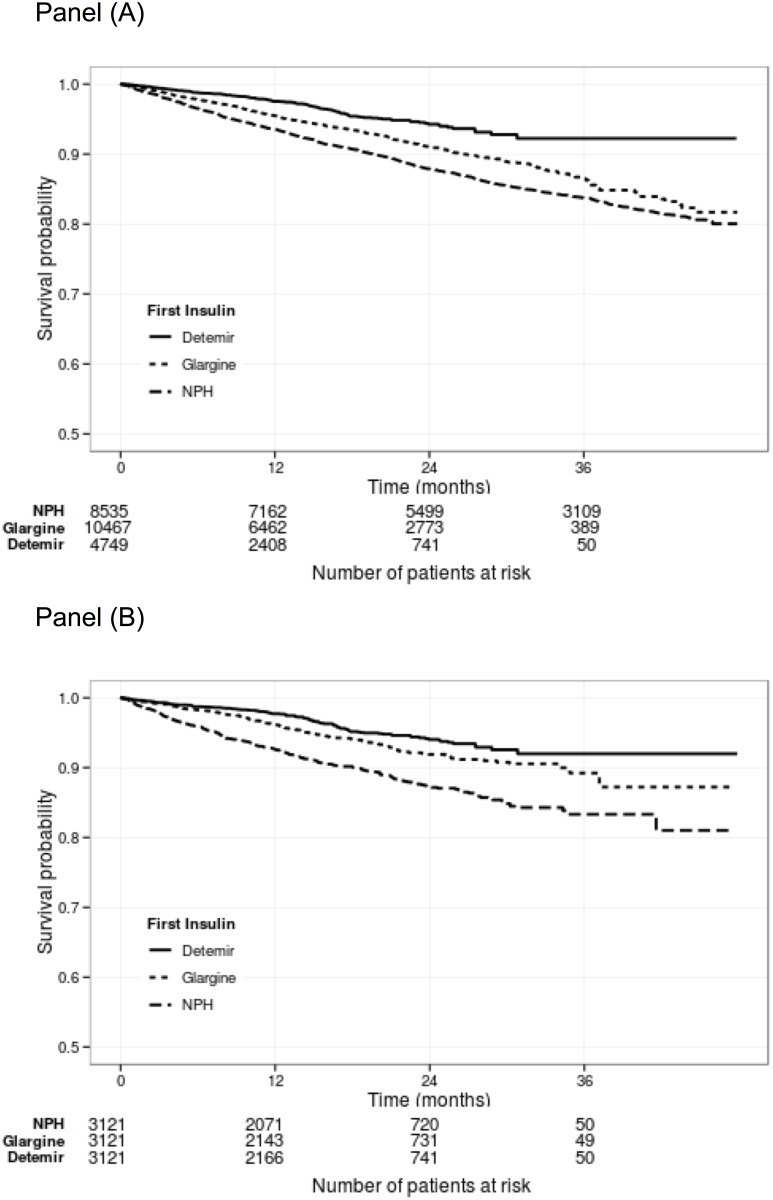
Kaplan-Meier survival curves for the initializers of the three basal insulins, Panel (A): unmatched cohort (n = 23 751), Panel (B): propensity score matched cohort (n = 9363), Log-rank p-values<0.001 for detemir vs. NPH, and glargine vs. NPH in both panels. Log-rank p-value for detemir vs. glargine <0.001 in Panel (A) and 0.005 in Panel (B).

Survival curves of the total cohort and the PS matched cohort are in Fig 2. Among the 23 751 patients there were 2078 deaths: 681 among new users of NPH, 149 of detemir and 556 of glargine. In the PS matched cohort of 9 363 patients, there were 620 deaths during the up to 4-year follow-up time (median 1.7, interquartile range 0.8 to 2.2 years). These deaths were divided as follows: During exposure to NPH: 183 deaths (absolute rate 55/1000 patient years, 95% CI 48 to 63), detemir: 90 deaths (absolute rate 22, 95% CI 18 to 27), and glargine: 146 deaths (absolute rate 31, 95% CI 26 to 37). Considering NPH as reference, the adjusted hazard ratio (HR) for detemir was 0.39 (95% CI, 0.30 to 0.50) and for glargine 0.55 (95% CI, 0.44 to 0.69). When compared to glargine, the adjusted HR for detemir was 0.71 (95% CI, 0.54 to 0.93) (Table 2).

**Table 2:** All-cause mortality estimated by Cox’s proportional hazards model using propensity score matched data. Reference category in brackets.

Variables (Reference category)	Categories	Hazard ratio	95% CI	P-value
Basal insulin (NPH)	Detemir	0.39	0.30, 0.50	<0.001
	Glargine	0.55	0.44, 0.69	<0.001
	Unknown*	3.12	2.52, 3.85	<0.001
Gender (Male)	Female	0.73	0.62, 0.86	<0.001
Age-group, (40–49 years)	50–59	1.17	0.72, 1.90	0.52
	60–69	2.16	1.38, 3.38	<0.001
	70–79	4.15	2.66, 6.47	<0.001
	80+	8.03	5.12, 12.59	<0.001
Prior use of non-basal** insulin at start of follow-up (No)	Yes	1.24	0.94, 1.62	0.12
Time dependent current use of non-basal** insulin during follow-up (No)	Yes	0.66	0.53, 0.82	<0.001
Prior use of sulpholnylurea at start of follow-up (No)	Yes	1.38	1.00, 1.91	0.05
Time dependent current use of sulpholnylurea during follow-up (No)	Yes	0.88	0.70, 1.11	0.29
Prior hospitalization due to severe hypoglycaemia at start of follow-up (No)	Yes	0.67	0.38, 1.20	0.18
Time dependent number of prior hospitalizations due to severe hypoglycaemia at start of follow-up (None)	1–2	1.75	1.03, 2.98	0.04
	≥3	2.51	1.16, 5.46	0.02
Time dependent switch of insulin during follow-up: purchase of other study insulin than the initiated study insulin (No)	Yes	1.73	1.32, 2.28	<0.001
Time from diagnosis (years) defined as time from first purchase of diabetes medication or time of reimbursement decision whichever occur first (≤1 year)	>1-≤2	1.00	0.66, 1.53	0.99
	>2-≤5	1.26	0.94, 1.69	0.13
	>5-≤10	0.83	0.60, 1.14	0.25
	>10	1.12	0.80, 1.59	0.51
Calendar year of purchase of first basal insulin, (2006)	2007	1.10	0.64, 1.88	0.74
	2008	1.15	0.66, 1.98	0.62
	2009	1.62	0.90, 2.90	0.11

* Category “Unknown” comprises the time periods where type of insulin was not available in database.

** “Prior use of non-basal insulin” denotes a prescription for a non-basal insulin.

There were 692 and 201 deaths in the unmatched, and matched cohorts respectively, during a period when the insulin type used could not be verified from registers. These deaths were taken into account using sensitivity analyses. (See S1 Table, Models 2 and 3.)

### Cause-specific mortality

In the PS matched cohort, cardiovascular diseases were the most frequent cause of death (N = 275, 44.4%), followed by cancer (N = 183, 29.5%), gastrointestinal diseases (N = 42, 6.8%), respiratory diseases (N = 29, 4.7%), and deaths of other causes (N = 91, 14.7%) (Table 3).

**Table 3:** Cause-specific mortality of users of detemir and glargine with insulin NPH as reference in the propensity score matched cohort.

Cause-specific mortality (ICD-10 diagnosis code)	Insulin NPH	Detemir			Glargine		
	N = 3121	N = 3121			N = 3121		
		Hazard ratio	95% CI	P-value	Hazard ratio	95% CI	P-value
Cardiovascular diseases (I00-I99)	1.0	0.42	0.28, 0.61	P<0.001	0.65	0.47, 0.91	P = 0.012
Cancer and neoplasms (C00-D48)	1.0	0.23	0.14, 0.40	P<0.001	0.35	0.22, 0.54	P<0.001
Digestive system (K00-K93)	1.0	0.45	0.19, 1.06	P = 0.064	0.44	0.19, 1.00	P = 0.049
Respiratory system (J00-J99)	1.0	0.18	0.04, 0.89	P = 0.036	0.76	0.28, 2.05	P = 0.59
Other causes of death	1.0	0.70	0.38, 1.30	P = 0.26	0.68	0.68, 1.27	P = 0.23

To account for multiple testing p-values smaller than 0.05/5 = 0.001 are considered statistically significant (Bonferroni correction).

Considering NPH as reference, the adjusted HR for detemir for cardiovascular mortality was 0.42 (95% CI, 0.28 to 0.61), and for glargine 0.65 (95% CI, 0.47 to 0.91). With exposure to glargine as reference the HR for detemir was 0.64 (95% CI, 0.43 to 0.95).

For cancer mortality the adjusted HR for detemir was 0.23 (95% CI, 0.14 to 0.40), and for glargine 0.35 (95% CI, 0.22 to 0.54) with NPH as reference. When compared to glargine, the HR for detemir was 0.67 (95% CI, 0.38 to 1.18).

For the deaths due to gastrointestinal diseases and with NPH as reference, the adjusted HR for both detemir and glargine tended to be reduced (0.45, 95% CI, 0.19 to 1.06; 0.44, 95% CI, 0.19 to 1.00, respectively).

### Sensitivity analyses

Among the PS matched population there were 3979 patients who had periods during which the type of insulin could not be verified in registers, equalling to 9% of the total follow-up time. During these periods of unknown exposure 201 deaths were recorded. The lack of information was typically due to hospitalization, or treatment in a nursing home, where insulin was supplied by the institution, and thus no prescription data was available from registers. Also, 5.5% of the cohort had previously used insulin for a short period, typically during an acute disease requiring treatment with a short-acting insulin. During follow-up 1130 patients switched their initial basal insulin treatment (825 from NPH, 219 from detemir, and 88 from glargine). We performed sensitivity analyses to account for these and other unmeasured confounders, and to estimate potential biases. (S1 Table) In these analyses the differences in HRs for mortality between the insulin types remained essentially unaltered. We found no evidence for interaction with gaps or switches of insulin use, or with cumulative exposure (measured in time). Moreover, the results were comparable for the total unmatched cohort. (S1 Table)

## Discussion

We found considerable differences in mortality risks related to the three basal insulins in a large, nationwide follow-up study of real-life patients in routine practice. The adjusted risk for all-cause mortality was 61% lower among users of detemir, and 45% lower for glargine, compared to the treatment with the conventional NPH insulin. Furthermore, detemir was associated with a significant risk reduction of 29% in comparison to glargine. Both cardiovascular and cancer related mortality were significantly higher in the NPH group.

A recent systematic review revealed a substantial lack of studies using hard endpoints such as cardiovascular mortality among users of different insulin regimen. [19] Also, we did not find any studies comparing cancer-related mortality between different insulins. Newer, longer acting basal insulins detemir and glargine have shown benefits over NPH in RCTs, but it has not been clearly demonstrated whether these advantages translate into lower mortality. Even long-term RCTs have a selected population, and they may not be representative of the general patient population and routine practice. Modern electronic patient records offer a source for studies comparing treatments in real-life settings, but these are difficult to conduct because of the many potential variables and changes in treatment over time.

### Strengths and limitations

In this regard, our study with patients from clinical practice has several strengths. We used national registers with reliable prescription and mortality data with a follow-up of 13 397 person-years in a propensity score -matched population. At the start of follow-up all participants were basal insulin-naïve. Additionally, our large database allowed adjustment for possible confounding factors, including switches and gaps in treatment. As demonstrated by the sensitivity analyses, the magnitudes of the adjusted HRs for mortality were robust and consistent both for the parent cohort of 23 751 participants as well as for the PS matched subcohort. Our observational study does not prove causality, but the adjusted and robust findings suggest more than just association.

A crucial question is whether the insulin groups were comparable at baseline. To reduce this potential bias we have used multiple statistical approaches. For example, the large database enabled the use of seven relevant covariates attributable to the choice of insulin by the prescriber. However, many important clinical variables were unavailable, and are thus not included in the PS model. For instance, we did not obtain information on lifestyle factors, body mass index, or comorbidities. Also clinical parameters such glucose, HbA1c levels, or renal function during the study are lacking. All these could potentially affect the choice of basal insulin by the prescribing physician. On the other hand, it is plausible that high risk patients would more likely be prescribed with longer acting insulin, which would drive our results towards null. For instance, the lower risk for hypoglycaemia shown in RTCs could have favoured the initiation of glargine or detemir for persons with cardiovascular disease, for whom hypoglycaemia could be anticipated to be more harmful. This could conversely benefit the NPH group in our study. Furthermore, the history of previous hospitalizations due to severe hypoglycaemia was taken into account in the adjusted analyses. Nevertheless, the possibility remains that physicians would have preferred the earlier generation insulin NPH to the newer analogues for some type of patients, leading to bias. However, this scenario would not clearly explain the significant differences we found between glargine and detemir. Also, the lack of data on concomitant oral medication for diabetes before or during the study period may constitute a bias. However, according to the national, evidence-based guidelines, generally only metformin is to be continued when add-on insulin treatment is initiated for patients with type 2 diabetes. Also, these guidelines did not give a preference for the type of insulin at initiation. Thus, we do not expect that differences, in for instance metformin use, would have guided the choice of insulin at baseline. We also lacked the information of insulin dosage and the participants’ body mass index during the study and were not able to examine dose–response relationships to support causal effects. However, these two parameters are apt to change, and they would be extremely difficult to control for in a study with real-life patients.

Second, detemir was the last to enter clinical use, and in our study there were only 55 detemir users in 2006, the number gradually increasing thereafter. This was taken into account by use of propensity scores. Furthermore, there was no difference in the availability or costs for the patient between the insulins during the study period. The gradual replacement of NPH with longer acting detemir and glargine in our study over the years 2006–2009 reflects the real-life situation in Finland and elsewhere.[3–4]

Third, the follow-up time was relatively short but in a real-life setting with changing treatment patterns it is difficult to sustain long periods of the same treatment. In our study population 14% of the patients switched the insulin first prescribed during the follow-up. By comparison, in a study of patients with type 2 diabetes treated in primary care in the UK approximately every fourth patient had changed the initial glargine and every third patient the initial NPH or detemir to some other preparation during a follow up of 24 months. [20]

Finally, there were 201 deaths in the PS matched population which occurred at a time when insulin use could not be verified by registers. This gap was typically associated with treatment in an institution, often at the end of life. The sensitivity analyses indicate that taking these deaths into account did not significantly change the results.

### Comparison with previous studies and possible explanations

Hypoglycaemia is the most common side effect of insulin therapy. [21] Several studies have reported an association between severe hypoglycaemia and mortality, especially in high risk patients. [22–23] Furthermore, large follow-up studies examining the benefits of stringent glucose control in type 2 diabetes–often pursued by using insulin therapy- have shown that while microvascular complications are reduced [24], the side effects of intensified treatment—especially hypoglycaemia and weight gain—may increase mortality. [25] However, most of the aforementioned studies have not specified the type of insulin therapy prescribed, which, in the light of our findings, merits further analysis. For comparison, in a study during 2001 to 2008, adding insulin as compared to adding sulpholnylurea to patients on metformin monotherapy was associated with 44% higher risk of all-cause mortality; a comparable difference to our finding between basal insulins. [26]

A difference in hypoglycaemia risk is a potential explanation for our results, especially as cardiovascular disease was the most frequent (44%) cause of death in our study. Compared to NPH, both longer-acting insulins, detemir and glargine, have been shown to provoke less hypoglycaemia.[9] In a previous study using Finnish national registers (N = 75 000), new users of detemir and glargine who were followed up for 4 years, had a 31% and 16% lower risk of a severe hypoglycaemic episode respectively.[14] We found a 58% lower risk of cardiovascular mortality for detemir, and a 35% lower risk for glargine. Of note, prior hospitalization due to severe hypoglycaemia was adjusted for in the PS matching.

Another possible explanation for our findings is a difference in weight gain among the patients with different basal insulins. Commencing insulin therapy often leads to an increase in body weight, which may be associated with cardiovascular disease, cancer and increased mortality. [27] Glargine, and especially detemir have been shown to cause less weight gain. [11,28] However, we lacked the information about weight changes during follow-up and were subsequently unable to account for this.

### Cancer related mortality

In our study, 30% of the deaths were due to cancer. The choice of insulin appeared to have an even greater impact on cancer related mortality than on cardiovascular mortality. In the adjusted analyses with NPH as comparator, detemir and glargine were associated with a 77% and 65% lower risk, respectively. Since the development of cancer takes many years, it seems extraordinary that we found a significant difference between the treatment groups in cancer related mortality during our follow-up of up to 4 years. However, a similar effect has been observed previously [29] suggesting that pre-existing malignant cells may rapidly proliferate into fulminant cancer when stimulated by the growth hormone action of insulin. Another hypothesis suggests that hyperglycaemia may play a role, whereby cancer growth could be delayed among those with well-controlled diabetes and preclinical cancer. [30] Thirdly, it has not been studied whether insulin therapy could negatively affect anti-cancer treatment thereby increasing mortality. We also analysed cancer mortality by censoring the first 12 months of insulin use (S1 Table, Model 10). This risk was 48% and 44% lower among detemir and glargine users, respectively, compared to NPH, but the number of events was small, and the finding was not statistically significant.

Although insulin studies with cancer mortality as an endpoint are few, insulin therapy has been associated with an increased cancer incidence. [31] In a German Insurance study of 127 000 patients, dose-adjusted use of glargine was associated with an increased risk for cancer compared with users of human insulin. [29] On the other hand, the randomized, controlled ORIGIN trial found no significant difference in cancer incidence between glargine and standard care during a follow-up of over 6 years. [32] Also, a record linkage study of over 70 000 patients in France comparing cancer incidence among new users of glargine, detemir, and human basal insulin did not find differences in risk. However, this study censored the first 6 to 12 months of exposure. [33] For detemir a possible association with cancer has not been extensively studied, but meta-analyses did not show any increase in cancer incidence during short RCTs. [34–35] In all, the large differences in cancer mortality in our study warrant randomised trials and may call for distinguishing the insulin type in older studies.

In conclusion, risk of all-cause, cardiovascular, and cancer mortality associated with insulin therapy in patients with type 2 diabetes, was significantly lower with detemir and glargine in comparison with NPH use. Overall, the choice of insulin may have more impact on the risks and benefits of intensive glucose treatment than has hitherto been considered.

## Supporting Information

S1 Table Sensitivity analysis for risk of all-cause mortality for the users of insulins detemir and glargine with NPH as reference. (DOCX)
