# Supplementary material for: All-Cause and Cause-Specific Mortality among Users of Basal Insulins NPH, Detemir, and Glargine
Source: PLoS One. 2016 Mar 31;11(3):e0151910. doi: 10.1371/journal.pone.0151910 (PMC4816506; doi:10.1371/journal.pone.0151910)
Supplement: S1 Table — (DOCX) [file pone.0151910.s001.docx]

# **Supporting information**

### Supporting information to:

Arto Y Strandberg, Fabian J Hoti, Timo E Strandberg, Solomon Christopher, Jari Haukka and Pasi Korhonen.

All-cause and Cause-specific Mortality among Users of Basal Insulin NPH, Detemir, and Glargine.

**S1 Table.** Sensitivity analysis for risk of all-cause mortality for the users of insulins detemir and glargine with NPH as reference.

| **Variables** | | **Detemir** | | **Glargine** | |
| --- | --- | --- | --- | --- | --- |
| **All-cause mortality** | | **Hazard ratio*** | **95% CI** | **Hazard ratio*** | **95% CI** |
| Model 1: Base model.  Follow-up periods with unknown exposure of study basal insulin form an unknown exposure category. | | 0.39 | (0.30, 0.50) | 0.55 | (0.44, 0.69) |
| Model 2:  Periods with unknown insulin exposure are censored. | | 0.39 | (0.30, 0.50) | 0.56 | (0.45, 0.71) |
| Model 3:  Assume use of last purchased basal insulin during periods of unknown insulin exposure. | | 0.39 | (0.32, 0.48) | 0.58 | (0.48, 0.69) |
| Model 4:  Time periods with overlapping use of insulin are censored. | | 0.38 | (0.29, 0.49) | 0.56 | (0.44, 0.70) |
| Model 5:  The follow-up period is censored at the purchase of an insulin different to the initial study insulin (switch). | | 0.36 | (0.28, 0.47) | 0.55 | (0.43, 0.69) |
| Model 6: Cumulative exposure of insulins model, with <1 year exposure to NPH as reference.  Follow-up time censored at 1^st^ switch of insulin. | < 1 year  1-2 years  > 2 years | 0.29  0.22  0.087 | (0.22, 0.38)  (0.14, 0.33)  (0.03, 0.25) | 0.53  0.23  0.13 | (0.42, 0.66)  (0.15, 0.35)  (0.06, 0.31) |
| Model 7: Total unmatched cohort (n= 23,751) | | 0.48 | (0.39, 0.58) | 0.62 | (0.55, 0.70) |
| Model 8:Total unmatched cohort analysis using the inverse of the propensity scores as weights (1% of the lowest and highest weights are truncated) | | 0.56 | (0.45, 0.69) | 0.68 | (0.59, 0.79) |
| Model 9: Patients with prior use of other insulin than study insulins at start of follow-up are removed | | 0.36 | (0.28, 0.48) | 0.53 | (0.42, 0.67) |
| Model 10: Cancer specific mortality with first 12 months of follow up censored. | | 0.52 | (0.18, 1.51) | 0.58 | (0.21,1.61) |

*All hazard ratios were significant (P<0.001) except for Model 10 where the P-value was 0.23 and 0.30, for detemir vs NPH and glargine vs. NPH, respectively.

**S1 Table explanation:**

Model 1:

In the base model we assume the use of at most one study basal insulin at a time (the most recent purchase). Periods with no/unknown exposure are classified into an “unknown exposure” category. In this model a person’s follow-up time is not censored at switch of study basal insulin.

Models 2 and 3:

As 201 deaths occurred during periods of unknown exposure, we performed two sensitivity analyses to the way we handled these periods. We performed a sensitivity analysis where follow-up periods of unknown exposure were censored (Model 2), and another with the assumption of extended use of the most recent insulin (Model 3).

Models 4, 5 and 6:

As a sensitivity analysis to our exposure definition, we performed an analysis where follow-up was censored at the first switch of the basal insulin (Model 4), an analysis where follow-up periods with overlapping use of insulin were censored (Model 5), and an analysis where cumulative duration of exposure to the insulin was considered (Model 6).

Models 7 and 8:

As a sensitivity analysis of the applied propensity score matching method, we repeated the analysis with the unmatched cohort (Model 7), and with the unmatched cohort with the inverse of propensity scores as weights (Model 8).

Model 9

Prior use of non-basal insulin consisted mostly of brief use of a short acting insulin.

The results remained largely unchanged in all models.
